# Supplementary material for: Heritability of the shape of subcortical brain structures in the general population
Source: Nat Commun. 2016 Dec 15;7:13738. doi: 10.1038/ncomms13738 (PMC5172387; doi:10.1038/ncomms13738)
Supplement: Supplementary Information — Supplementary Figures 1-5, Supplementary Table 1 [file ncomms13738-s1.pdf]

Supplementary Figure 1 | Correlation of heritability estimates between basic model and model with additional intracranial volume adjustment.

Supplementary Figure 2 | Dependency between effect on heritability estimates and correlation with structure specific volume.

Figure Legend: Figure shows difference between vertices heritability of basic model and model additionally adjusted by structure specific volume (shown in red), compare to correlation of shape measurement with structure volume (shown in green) and the shape measurement itself (shown in blue). Vertices on x-axis are sorted from low to high correlation with structure volume.

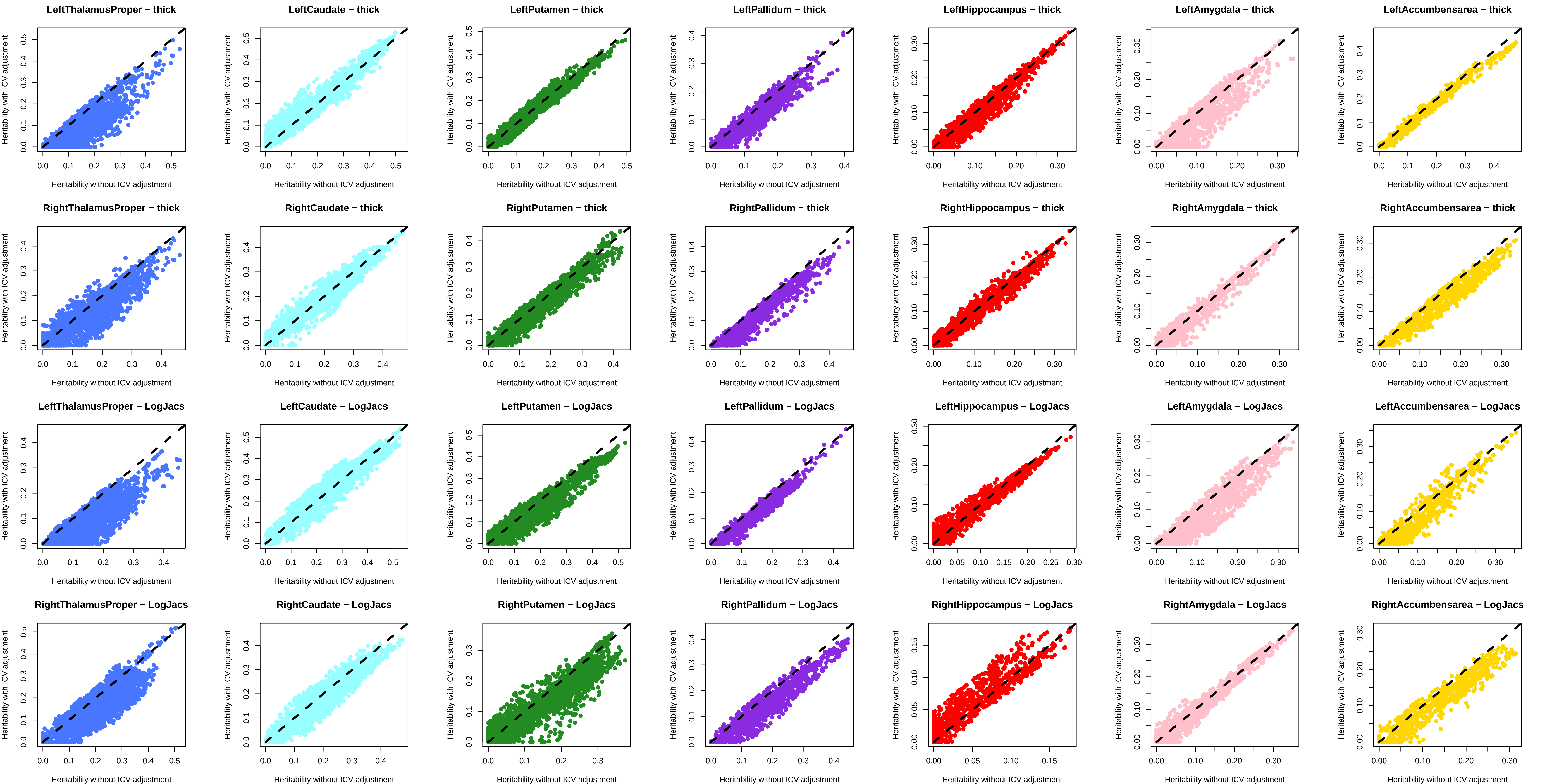
LeftThalamusProper − thick
LeftCaudate − thick
LeftPutamen − thick
LeftPallidum − thick
LeftHippocampus − thick
LeftAmygdala − thick
LeftAccumbensarea − thick
RightThalamusProper − thick
RightCaudate − thick
RightPutamen − thick
RightPallidum − thick
RightHippocampus − thick
RightAmygdala − thick
RightAccumbensarea − thick
LeftThalamusProper − LogJacs
LeftCaudate − LogJacs
LeftPutamen − LogJacs
LeftPallidum − LogJacs
LeftHippocampus − LogJacs
LeftAmygdala − LogJacs
LeftAccumbensarea − LogJacs
RightThalamusProper − LogJacs
RightCaudate − LogJacs
RightPutamen − LogJacs
RightPallidum − LogJacs
RightHippocampus − LogJacs
RightAmygdala − LogJacs
RightAccumbensarea − LogJacs
Heritability with ICV adjustment
Heritability without ICV adjustment

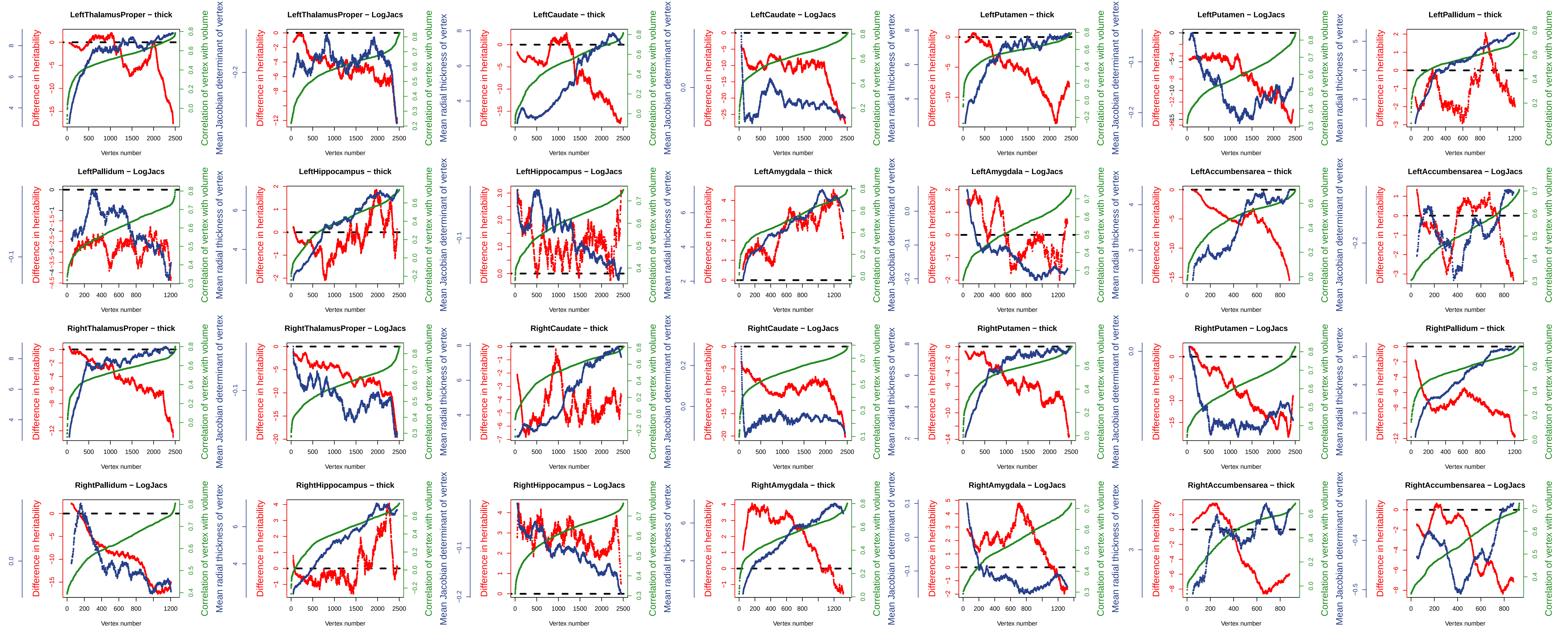

LeftThalamusProper – thick
LeftThalamusProper – LogJacs
LeftCaudate – thick
LeftCaudate – LogJacs
LeftPutamen – thick
LeftPutamen – LogJacs
LeftPallidum – thick
LeftPallidum – LogJacs
LeftHippocampus – thick
LeftHippocampus – LogJacs
LeftAmygdala – thick
LeftAmygdala – LogJacs
LeftAccumbensarea – thick
LeftAccumbensarea – LogJacs
RightThalamusProper – thick
RightThalamusProper – LogJacs
RightCaudate – thick
RightCaudate – LogJacs
RightPutamen – thick
RightPutamen – LogJacs
RightPallidum – thick
RightPallidum – LogJacs
RightHippocampus – thick
RightHippocampus – LogJacs
RightAmygdala – thick
RightAmygdala – LogJacs
RightAccumbensarea – thick
RightAccumbensarea – LogJacs
Vertex number
Difference in heritability
Correlation of vertex with volume
Mean radial thickness of vertex
Mean Jacobian determinant of vertex

Supplementary Figure 3 | Reproducibility maps of shape measures of subcortical brain regions.

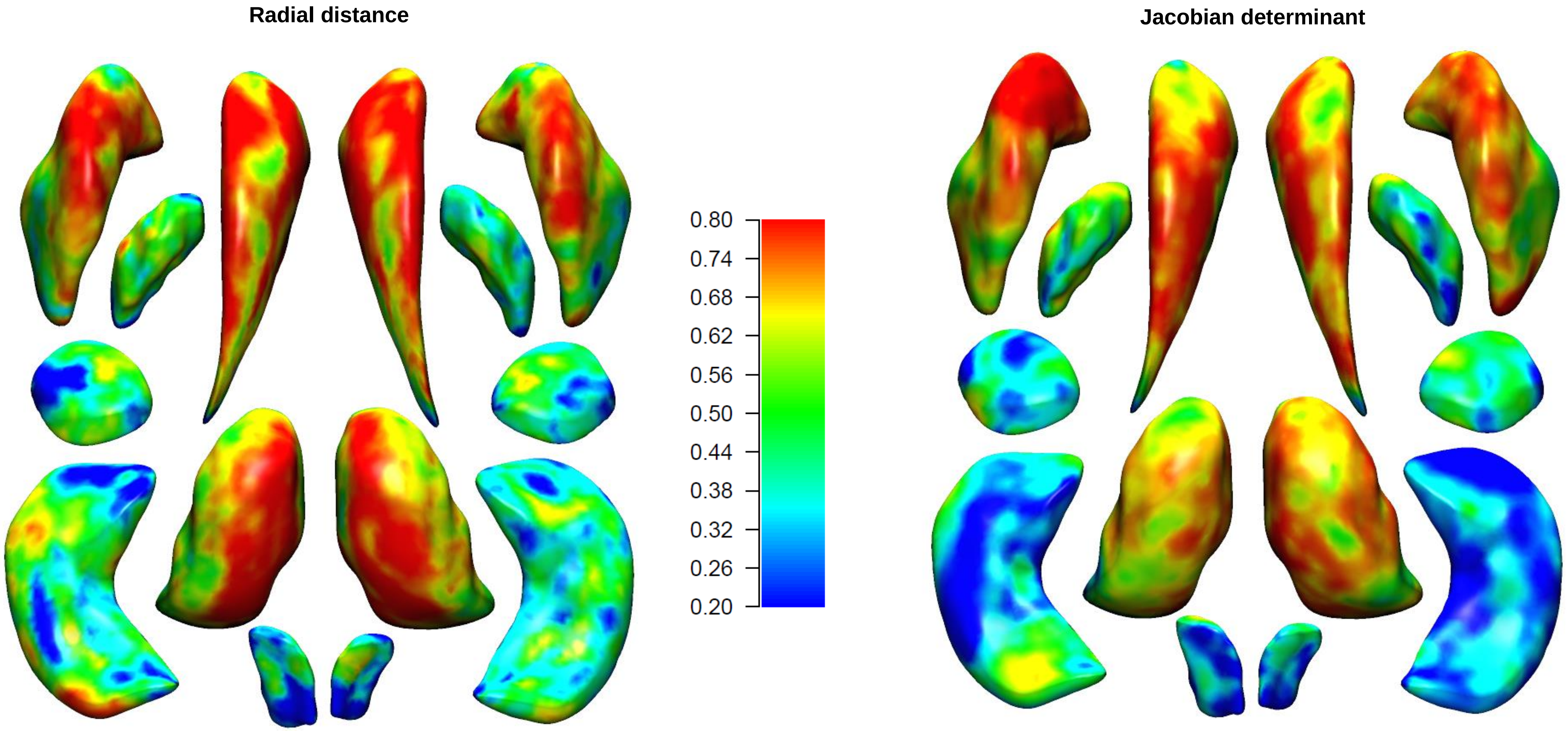

Supplementary Figure 4 | Heritability maps of shape measures of subcortical brain regions under various models for QTIM.

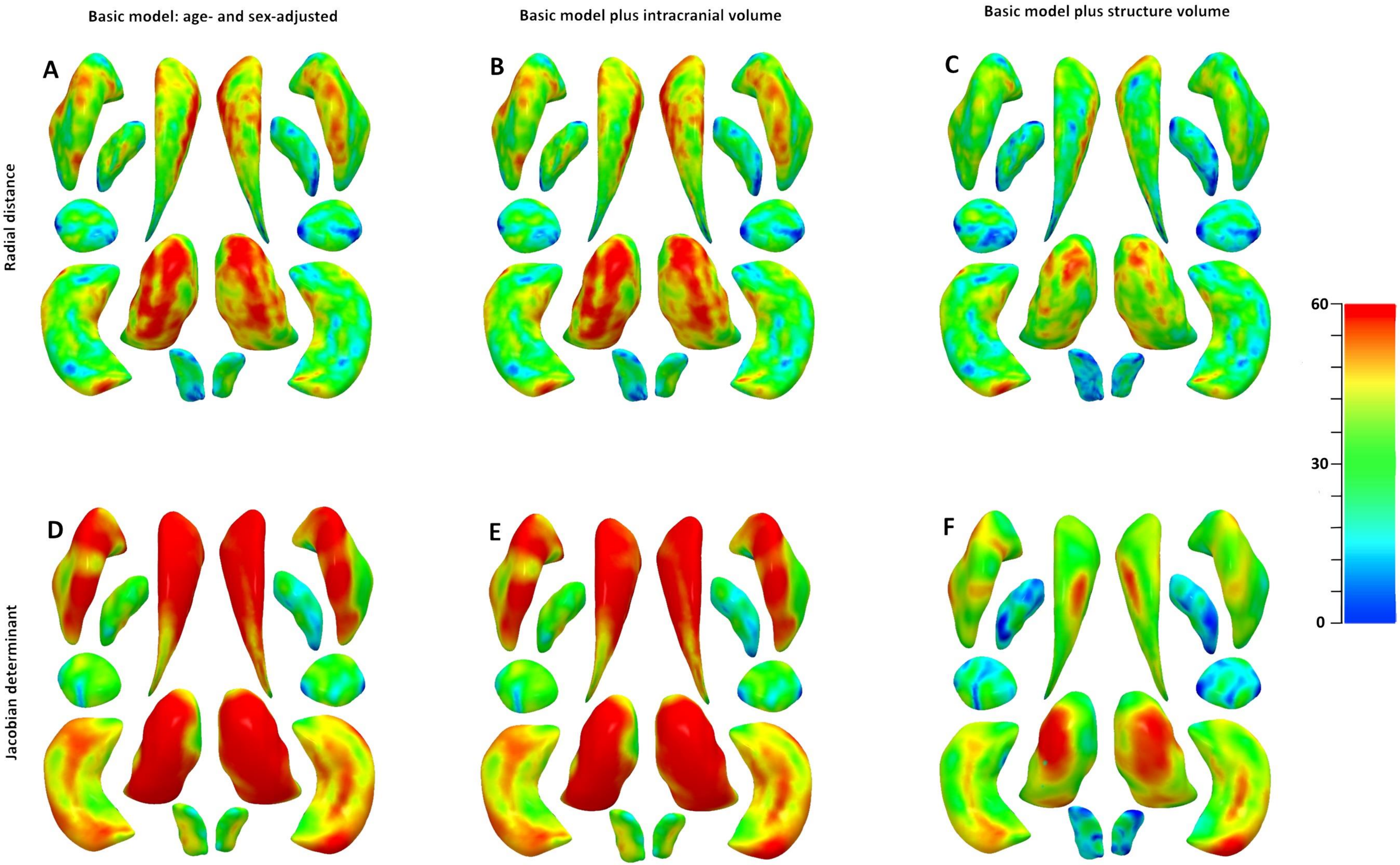

Supplementary Figure 5 | Concordance between the Rotterdam Study and QTIM heritability of subcortical shape. The correlation of the twin-based and population-based estimates was high (Pearson's correlation coefficient = 0.28, p = 3.03 ×10^{-306}).

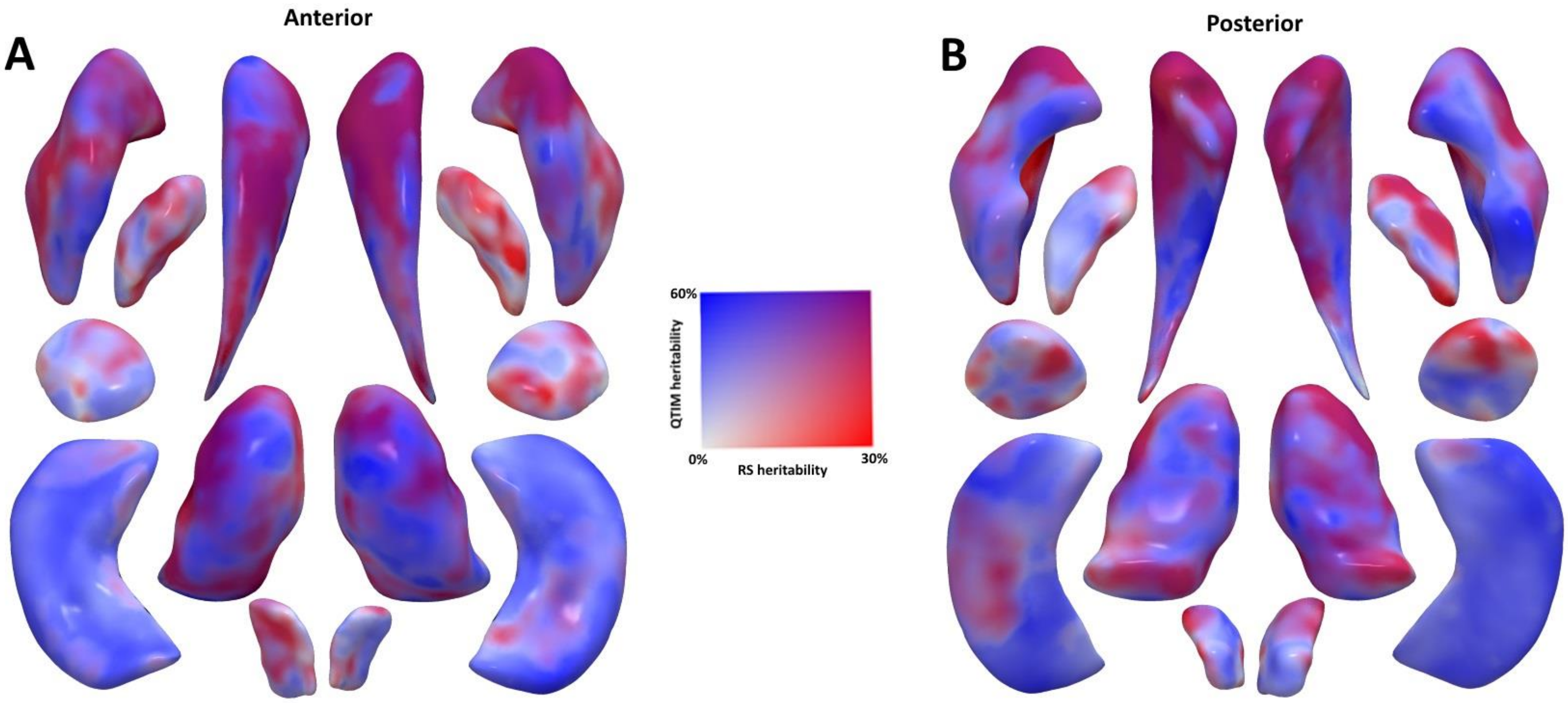

Supplementary Table 1| Correlation between the order of principal components based on their eigenvalues with the order based on their heritablity estimates.

Shape measure	Subcortical structure	Correlation coefficient	Correlation p-value
Jacobian determinant	LeftAccumbensarea	0.09616054	0.003331576
Jacobian determinant	LeftAmygdala	0.069908572	0.009696363
Jacobian determinant	LeftCaudate	0.027834067	0.163972728
Jacobian determinant	LeftHippocampus	-0.037756799	0.058982403
Jacobian determinant	LeftPallidum	0.088463309	0.001714587
Jacobian determinant	LeftPutamen	0.026317332	0.188186482
Jacobian determinant	LeftThalamusProper	0.049058832	0.014120862
Jacobian determinant	RightAccumbensarea	-0.028269652	0.389171322
Jacobian determinant	RightAmygdala	0.059445261	0.027905966
Jacobian determinant	RightCaudate	-0.003630907	0.855953315
Jacobian determinant	RightHippocampus	0.009677033	0.628517451
Jacobian determinant	RightPallidum	0.047227114	0.094590398
Jacobian determinant	RightPutamen	-0.011560145	0.563285224
Jacobian determinant	RightThalamusProper	0.041235252	0.039166474
Radial thickness	LeftAccumbensarea	0.00374144	0.909280971
Radial thickness	LeftAmygdala	0.063005545	0.019777678
Radial thickness	LeftCaudate	0.001802509	0.928194641
Radial thickness	LeftHippocampus	0.028278783	0.15733825
Radial thickness	LeftPallidum	0.09619236	0.00064757
Radial thickness	LeftPutamen	0.02941723	0.141282254
Radial thickness	LeftThalamusProper	0.049494386	0.013286755
Radial thickness	RightAccumbensarea	-0.037185062	0.257273619
Radial thickness	RightAmygdala	0.022838592	0.398637481
Radial thickness	RightCaudate	0.017432371	0.383427938
Radial thickness	RightHippocampus	-0.006370233	0.750118573

Radial thickness	RightPallidum	0.023218884	0.411353161
Radial thickness	RightPutamen	0.007882656	0.693507568
Radial thickness	RightThalamusProper	0.076328208	0.000132586
